# Hematoma Expansion in Intracerebral Hemorrhage: An Update on Prediction and Treatment

**DOI:** 10.3389/fneur.2020.00702

**Published:** 2020-07-17

**Authors:** Zhifang Li, Mingfeng You, Chunnan Long, Rentang Bi, Haoqiang Xu, Quanwei He, Bo Hu

**Affiliations:** Department of Neurology, Union Hospital, Tongji Medical College, Huazhong University of Science and Technology, Wuhan, China

**Keywords:** hematoma expansion, intracerebral hemorrhage, risk factor, prediction score model, treatment

## Abstract

Intracerebral hemorrhage (ICH) is the most lethal type of stroke, but there is no specific treatment. After years of effort, neurologists have found that hematoma expansion (HE) is a vital predictor of poor prognosis in ICH patients, with a not uncommon incidence ranging widely from 13 to 38%. Herein, the progress of studies on HE after ICH in recent years is updated, and the topics of definition, prevalence, risk factors, prediction score models, mechanisms, treatment, and prospects of HE are covered in this review. The risk factors and prediction score models, including clinical, imaging, and laboratory characteristics, are elaborated in detail, but limited by sensitivity, specificity, and inconvenience to clinical practice. The management of HE is also discussed from bench work to bed practice. However, the upmost problem at present is that there is no treatment for HE proven to definitely improve clinical outcomes. Further studies are needed to identify more accurate predictors and effective treatment to reduce HE.

## Introduction

Intracerebral hemorrhage (ICH) accounts for about 10–20% of all types of stroke (1, 2) and almost 40% of patients who suffer from ICH will die within the first month, while only 12–39% of survivors achieve long-term functional independence (2), which makes it a severe public health problem. Unfortunately, after decades of effort there is no specific treatment yet for ICH, but recently HE has been found to be a modifiable and independent predictor of clinical neurological deterioration in intracerebral hemorrhagic patients. HE prevention has been accepted as one of the most promising therapeutic strategies in ICH treatment (3). Although numerous efforts have been made to select ICH patients at high risk of developing HE, no uniform prediction score model can be concluded from current studies, which therefore impedes the early detection and subsequent active intervention by clinicians. Moreover, once HE occurs in ICH patients, the treatment is extremely limited and the functional prognosis of those patients is unsatisfactory. Thus, it remains a priority to prospectively detect high-risk HE patients as well as administer more active prevention treatment.

This review refers to the definition, prevalence, risk factors/predictors, prediction score models, mechanisms, and treatment progress of HE. In detail, the predictors of HE are summarized to update the view on the prediction, and suggestions are also provided to establish an accurate and easy-to-use prediction score model. Furthermore, we discuss current HE treatment strategies from the viewpoint of clinicians and also point out future directions for the discovery of more effective therapies.

## Definition

HE is defined based on visually discernible hematoma volume changes between the baseline and follow-up CT, and the evaluation of hematoma volume growth is diverse across HE related studies. As one of the earliest researchers of HE, Fujii et al. defined it as absolute hematoma volume growth of more than 20 ml or relative hematoma volume growth of more than 50% (4). Later, Brott et al. used relative hematoma volume growth of more than 33% which can be discovered by CT to define HE (5). Using ROC curve analysis, Kazui et al. applied cut point of hematoma volume increase of 12.5 ml or 40% (6). Some recently published large clinical trials also used relative hematoma growth of more than 33% (7), or combined hematoma growth of 6 mL absolute increase and 33% relative increase (8, 9) to define it. Based on CT angiography contrast extravasation, a 6 mL absolute increase of hematoma volume was proposed in the studies of Thompson et al. and Delgado et al. (10, 11). Of all the different definitions, Dowlatshahi et al. found that absolute growth definitions were more preferable to predict the outcome of ICH (12). Considering that intraventricular hemorrhage (IVH) is a predictor of poor prognosis in ICH, the presence of IVH may also be an indicator of HE. Specifically, Vignan et al. found that addition of IVH into the HE definition improves the prediction of 90 days outcome in ICH patients (13).

A uniform definition of HE considering convenience of measurement and effectiveness in predicting outcome is needed for further study.

## Prevalence

Hemostasis was once thought to be over within min after ICH occurrence, but recently HE has been found to be a common phenomenon of ICH with advanced radiology (5). The reported incidence of HE within 6 h from ICH symptom onset ranges widely from 13 to 38% (5, 6, 12), which may be largely explained by different definitions of HE and different time interval between hematoma measurement in the different studies. In the Intensive Blood Pressure Reduction in Acute Cerebral Hemorrhage Trial 2 (INTERACT2), the incidence of HE was 33.1% in the control group (14), while it was 25.3% in Antihypertensive Treatment of Acute Cerebral Hemorrhage 2 (ATACH2) (15). Notably, the incidence of HE is highest at the hyperacute stage (within 6 h after symptom onset) and HE usually occurs at the internal capsule, thalamus, and brainstem. Thus, it is necessary to maintain hematoma surveillance, especially at the hyperacute stage of ICH.

## Risk Factors/Predictors

ICH patients with high risk factors of developing HE are predisposed to experience clinical deterioration and closer neurological monitoring is required, while the absence of the predictors may identify ICH patients with low risk of developing HE. Thus, HE predictors have a vital role to play in selecting high-risk ICH patients and subsequently facilitating the individualized treatment. Based on clinical, imaging, and laboratory characteristics, a series of risk factors/predictors of HE have been identified.

## Clinical Predictors

Systolic blood pressure (SBP) is positively related to the initial hematoma volume in ICH patients (16) and the risk of HE is much higher in patients with post-admission SBP over 160 mmHg (*P* = 0.0074) (17, 18), which may be partly explained by the continuous rupturing and hemorrhaging of small vessels, thus making early blood pressure a potential treatment target. Although baseline blood pressure variability (BPV) is not associated with HE (19), post-admission BPV independently predicts HE as well as poor functional outcomes (20). High mean arterial pressure (MAP) is positively related to HE as well (19).

Medication with antiplatelet or anticoagulant drugs also increases the risk of HE. In high-income countries, more than a quarter of patients with ICH are on prior antiplatelet therapy (APT) (21). An observational study by Toyoda et al. showed that APT was an independent predictor of HE (22), while in the Cerebral Hemorrhage and NXY-059 treatment trial, antiplatelet drug use at ICH onset was not associated with HE (23). On account of their methodological difference, recently this controversy has been laid to rest by a meta-analysis which supports prior APT as a predictor of HE (24). For those with prior medication with anticoagulants, prior oral anticoagulation (OAC) use is not only an independent predictor of larger initial hematoma volume (25) but also increases the risk of HE 6.2 times (26). In contrast to OAC, the incidence of ICH in Non-Vitamin K oral anticoagulants (NOACs) patients is dramatically decreased (27). NOACs-ICH has a lower risk of developing HE and is associated with smaller baseline hematoma volume (28) and better functional outcomes (29–31).

Higher baseline NIHSS or GCS scores (32–34), elevated body temperature (35), baseline weight (36), and history of cerebral infarction (37) or alcohol abuse (38) may increase the risk of HE, as found by some observational studies, and further randomized trials are needed to determine their relevance. In a recent retrospective cohort study of ICH patients with liver fibrosis, fibrosis-4 score and Aspartate Aminotransferase-Platelet Ratio Index were associated with HE (39).

Gender (40) and age (41) are also associated with HE, as men and older subjects (age ≥85 years) are more likely to present HE than women and younger subjects (40, 41).

It is interesting to note that ICH occurring during the daytime tends to be more likely to present HE than when occurring at night (OR, 3.53) (42). In addition, HE is mostly found in early initial CT scan (≤3 h of onset) (5, 6, 43), so time interval from ICH onset to initial CT scan should be considered (Abovementioned clinical predictors are summarized in Table 1).

**Table 1:** Clinical predictors of HE.

**Clinical features**	**References**	**Country**	**Sample size**	**Baseline CT time**	**Follow-up CT time**	**Definition of HE**
SBP	Ohwaki et al. (18)	Japan	76	At admission	The day after admission	Absolute growth > 12.5 mL, or relative growth of > 40%
Prior APT	Toyoda et al. (22)	Japan	251	At admission	24 h later after admission	Relative growth of > 40%
Prior VKAs	Flibotte et al. (26)	America	183	At admission	Up to 7 days after admission	Relative growth of > 33%
Prior NOACs	Takahashi et al. (30)	Japan	78	At admission	Within 24 h after baseline	–
Gander	Marini et al. (40)	America	2212	Within 6 h of onset	Within 24 h after baseline	Absolute growth > 6 mL, or relative growth of > 33%
Age	Forti et al. (41)	Italy	383	Within 6 h of onset	Ranging 4 36 h after baseline	Absolute growth > 6 mL, or relative growth of > 33%
Time from ICH symptom onset	Kazui et al. (6)	Japan	204	Within 48 h of onset	Within 120 h of onset	Absolute growth > 12.5 mL, or relative growth of > 40%
Day-night variability	Yao et al. (42)	America	111	Within 3 h of onset	Within 75 h of onset	Absolute growth > 6 mL or relative growth of > 33%

## Imaging Predictors

Some imaging phenomena that have been described in association with HE may have their origin in the pathophysiological processes around the hematoma, caused, e.g., by the breakdown of the blood brain barrier after ICH, which reflects the infiltration of blood into the peri-hematoma tissues and secondary damages resulting from the blood component such as albumin.

### CTA Predictors

Based on increased penetration of contrast agent, Wada et al. first described the spot sign (44), a single or multiple focus of contrast enhancement in the hematoma, which is considered a risk factor of death and clinical neurological deterioration (45) and an independent predictor of HE with 51–62% sensitivity and 85–88% specificity in different clinical research centers (8, 45). Intriguingly, the numbers of spot sign predominantly determine the value of spot sign in predicting HE (46), while the positive predictive value of spot sign is inversely associated with ICH onset-to-CTA time, which may indicate the dynamic process of spot sign (43). With further studies, some modified spot signs of CT perfusion (CTP), venous phase CTA, post contrast CT (PCT), and 90-s delayed CTA have presented with higher sensitivity and specificity (47, 48). Considering the resemblance of blood vessels in the hematoma and spot sign, Yi et al. found that continuous CTA source images could exclude blood vessels and improve its accuracy in predicting HE (49).

The leakage sign and higher Iodine Concentration (IC) within spot sign have been found to increase its sensitivity and specificity. IC was demonstrated to be an important characteristic of the spot sign and combining higher IC (i.e., IC > 7.82,100 μg/ml) with spot sign was an independent predictor of HE with sensitivity of up to 0.81 (50). The leakage sign which refers to a 1 cm diameter region of interest (ROI) and an increase of more than 10% in high Hounsfield unit (HU) in the ROI was proposed by Orito et al. and showed significantly higher sensitivity (93.3%) and specificity (88.9%) for predicting HE (51).

CTA spot sign and leakage sign can easily predict the HE, and CTA seems to be a good screening tool in detecting the secondary cause of the bleeding, the vascular malformations in ICH patients. However, its clinical practice is actually restricted by limited use of CTA, and there is a pressing need to discover more convenient imaging predictors in ICH patients.

### Non-contrast Computed Tomography (NCCT) Predictors

There are three categories of NCCT predictors: the large initial volume, irregular shape, and heterogeneity of the hematoma (52). The first two are well-known for predicting HE (52–55). Density heterogeneity is more and more noteworthy nowadays and some signs have been discovered to suggest HE.

**Figure 1:**
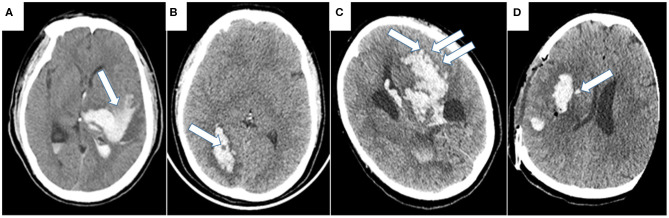
Representative examples of NCCT markers of HE. **(A)** Blend sign refers to the blending of hyperattenuating region with adjacent relatively hypoattenuating area with a well-defined margin. **(B)** Black hole sign refers to the round, oval, or rod-shaped relatively hypoattenuated area inside the hyperattenuated hematoma with a clear border to nearby brain tissue. **(C)** Island sign refers to three or more scattered small hematoma detached from the main hematoma, or more than 4 small hematoma connected partly or wholly with the main hematoma. **(D)** Satellite sign refers to the high-density dots around the hematoma.

Blend sign (Figure 1), the blending of the hyperattenuating region with an adjacent relatively hypoattenuating area with a well-defined margin, was first found by Li et al. which is easy to use for predicting HE (56, 57) and poor functional outcome (58) with 95.5% specificity. Black hole sign (Figure 1), the round, oval, or rod-shaped relatively hypoattenuated area inside the hyperattenuated hematoma with a clear border with nearby brain tissue, is another NCCT predictor with 94.1% specificity (59), which presents predictive accuracy for HE but is not an independent predictor of poor outcome compared with other NCCT features (7, 59, 60). The island sign (Figure 1), three or more scattered small hematoma detached from the main hematomas, or more than 4 small hematomas connected partly or wholly to the main hematoma (61), is not only an appropriate shape-related predictor for HE, with 98.2% specificity, but also a novel imaging marker to predict long-standing poor prognosis (61–63). However, the three abovementioned NCCT signs presented with disappointing sensitivity (39.3% of blend sign, 31.9% of black hole sign, and 44.7% of island sign), which dramatically decreases their practical clinical value. Later, satellite sign (Figure 1), which refers to high-density dots around the hematoma, was first put forward by Shimoda et al. and was demonstrated to be associated with large hemorrhage size (64). Recently, ICH patients presenting with high hematoma sedimentation levels, which may result from insufficient hemostasis, were predisposed to worse outcome in the study of Sato et al. (65). Other NCCT signs, such as hypodensities and the swirl sign (isodensity or hypodensity within a hyperdense area extending across 2 consecutive 5 mm axial CT sections), have also been proposed as predictors of HE (66, 67), but future clinical trials are needed to validate their predictive value in clinical practice. In addition to the descriptive signs, other researchers use quantitative methods to accurately measure the HU within the hematoma. Jeong et al. recently revealed that lower mean HU of a hematoma, which may be a clue of impaired clot contraction, is more likely to present HE (68). When minimal CT attenuation value in the hematoma is <31 HU, the ICH patients is more likely to develop HE (69).

Before interpreting these NCCT predictors, there are several drawbacks to be found in the studies: (1) the definitions of HE are diverse; (2) most of the studies are derived from a small sample size in single center cohorts, which may increase the risk of selection bias; (3) several ICH patients, such as those with oral anticoagulant treatment, are excluded from enrollment narrowing clinical application. Thus, the use of these predictors in clinical settings warrants further multi-center studies with larger samples and uniform criterion to validate their predictive value. Moreover, the above-mentioned NCCT signs actually share similar features in identifying the physiopathologic changes in HE, and if we can propose combined characteristics by integrating the terminology and diagnostic criteria of the NCCT predictors, it would be significant for predicting HE. Based on this hypothesis, an NCCT radiomics model, which was established from high-dimensional quantitative features of NCCT images, exhibited better predictive value on HE than a radiological model (70).

Although a few studies have compared the predictive effect of NCCT markers and CTA predictors (71, 72), we cannot conclude a distinct superiority of one predictor over the others due to their great heterogeneity in HE definition, terminology, and CTA acquisition protocol. In addition, even if there are several imaging predictors which are feasible for clinical practice, their application is largely constrained by lower sensitivity. Hence, a prediction score model combining two or more NCCT predictors, or clinical predictors or laboratory markers may increase the sensitivity and match the demand of clinical practice.

### Magnetic Resonance Imaging (MRI) Markers

In clinical settings, ICH patients usually undergo MRI for etiological evaluation. In 1998, Murai et al. first reported MRI spot sign in 108 ICH patients, which refers to the contrast extravasation within the hematoma. They found that MRI spot sign was closely associated with HE evidenced by follow-up CT scans (73). However, in a recent prospective study of 50 ICH patients, MRI spot sign failed to have statistical significance in predicting HE (74). In addition to the conflicting results on HE prediction, it takes a long time to conduct an MRI scan and it is relatively expensive. Thus, the value of MRI imaging in predicting HE is limited.

## Laboratory Predictors

### Coagulation Status

In general, alteration of coagulation function is significant in those with coagulation dysfunction due to blood system diseases and antiplatelet or anticoagulant drugs use, not hypertension ICH patients; therefore, attention should be specifically paid to the former. In those patients, elevated D-dimer (D-D) level, decreased fibrinogen, and international normalized ratio (INR) >1.5 are found to be predictors of HE in multivariate analysis (32, 38). Moreover, coagulation factor deficiency due to liver dysfunction also increases the risk of developing HE (75, 76).

### Blood Glucose

More than 50% of stroke patients present with admission hyperglycemia (77), a result of abnormal energy metabolism in response to ICH at the acute stage. However, admission hyperglycemia is conversely correlated with poor functional outcomes and high fatality rates regardless of diabetes history (78, 79). Some recent observational studies validated that admission hyperglycemia is related to the imaging predictors of HE, including island sign, spot sign, and blend sign (80–82). Moreover, a *post-hoc* analysis by Qureshi et al. in indicated that admission hyperglycemia increased the risk of developing HE 2.5-fold (83), which may be mediated by plasma kallikrein (84).

### Inflammation and Microvascular Integrity Markers

Inflammation response to ICH is an important factor for developing HE, which may lead to progressive damage of peri-hematoma vessels and continuation of bleeding. More research is revealing that high plasma concentrations of inflammatory factors, such as IL-6 (>24 pg/ml), CRP (>10 mg/L), and cellular fibronectin (c-Fn > 6 μg/ml), are associated with HE (85–87). Specifically, the risk of HE in patients with high plasma concentrations of CRP, IL-6, and c-Fn, respectively, increase 4-, 16-, and 92-fold, respectively, compared with normal counterparts (85). Moreover, the elevated matrix metalloproteinase-9 (MMP-9) level, an important cause of blood brain barrier (BBB) breakdown in acute cerebrovascular events, is also an independent risk factor for HE (OR value 15.65) (85, 88).

### Others

Low level of serum cholesterol, calcium, hemoglobin, or magnesium and high serum creatinine levels have also been reported to be correlated with HE in observational studies (36, 80, 89–93). In addition, variants of apolipoprotein E (APOE) predispose patients with lobar ICH to HE (94). As most of these biochemical indicators are commonly tested in clinical settings, the use of these potential predictors may largely facilitate clinical practice but further clinical trials are warranted.

## Prediction Score Model

As the low sensitivity or specificity of one predictor limits the clinical practice of these predictors, combining more predictors to set up a prediction score system may be a solution to the problem. Based on univariate and multivariable logistic regression analysis, several prediction score systems have been established to predict the risk of HE (Table 2).

**Table 2:** Comparison of prediction score models.

	**Creator**	**Time**	**Country**	**Sample size**	**C-statistic**
3-predictor model	Ririko et al.	2013 Jul	Japan	201	-
9-point score	Brouwers et al.	2014 Feb	America	817	0.72, 0.77*
24-point score	Wang et al.	2015 Feb	21 countries	964	0.73
PREDICT A/B score	Huynh et al.	2015 Nov	6 countries	301	–
HEP score	Yao et al.	2015 Oct	China	237	0.76
Basal ganglia score	Huang et al.	2017 Dec	China	266	–
BAT score	Morotti et al.	2018 May	Several countries	344	0.77, 0.70*
HEAVN scale	Miyahara et al.	2018 Sep	Japan	622	0.81, 0.80*
4-predictor model	Rustam et al.	2018 Oct	–	–	0.78
5-predictor model	Rustam et al.	2018 Oct	–	–	0.83
NAG scale	Sakuta et al.	2018 Dec	Japan	118	0.81

* *The former statistic for the development cohort, the latter statistic for the validation cohort*.

Based on clinical and imaging predictors, a practical prediction model was created comprised of hematoma volume, hematoma heterogeneity, and systolic BP 1.5 h after admission. However, further clinical trials are needed to validate its prediction value (95). Brouwers et al. later established a 9-point prediction score on four predictors: warfarin use, spot sign, time from ICH onset to the initial computed tomography, and baseline ICH volume, which demonstrated strong association with HE (OR, 4.59) (96). Although prospectively collected data and large sample size in the study added robustness to the results, a high dropout rate of patients with prior warfarin use or large baseline hematoma volume led to an underestimation of the predictive ability. Considering that CTA is not always available in acute ICH, a 24-point score, which is derived from sub-studies of INTERACT1 and 2, removed CTA spot sign and added intraventricular hemorrhage (IVH) extension and recurrent ICH (97). Despite both 9-point and 24-point scores showing acceptable discrimination, their calibration remains to be improved. Two new scores, the PREDICT A and B score, were then created substituting GCS or NIHSS score for baseline ICH volume and showed improved discrimination (32). It is worth noting that the PREDICT scores are suited only to supratentorial ICH because infratentorial ICH was excluded and the cohort had a relatively small sample size; thus, they require further independent validation. Another new Hematoma Expansion Prediction (HEP) score added a history of dementia and smoking and showed satisfactory discrimination ability (C-statistics, 0.76) (98). Based on multivariable logistic regression analysis, a basal ganglia score adopted three NCCT markers (island sign, blend sign, and swirl sign) and demonstrated reliable accuracy in predicting HE (*P* < 0.001) (99). Furthermore, to further simplify the prediction at the bedside, three new scales (the BAT score, NAG scale, and HEAVN scale) which added more practical predictors have been established from retrospective studies and have shown acceptable sensitivity and specificity (33, 34, 100, 101), but prospective validations of these scores are warranted. In a patient-level meta-analysis, Al-Shahi et al. analyzed predictors from diverse cohorts with large sample size to develop HE prediction models using four (time from symptom onset to CT, baseline ICH volume, antiplatelet use, and anticoagulant use) or five predictors (with the addition of spot sign). Both prediction models were externally validated and showed good discrimination (102).

Although many prediction scores have been proposed, currently none of them can impact clinical decision-making. First, they are inconvenient in clinical practice and it is difficult for clinicians to fulfill the score in a short time. Second, most of the predictors in these scores were derived from retrospective studies and only a few scores were demonstrated in external prospective trials, which implies that their accuracy remains to be examined. Third, the majority of the studies collected date from small-sized cohorts in one single center, which increases the risk of selection bias and decreases their accuracy in predicting HE. In the future, scales combining convenience and accuracy should be established and confirmed in large external and prospective studies, which will greatly contribute to clinical HE prediction.

## Pathophysiology

HE was once conceptualized as a continuing or recurrent bleeding partly caused by coagulation dysfunction or hemodynamic instability, until Miller Fisher proposed an alternative “avalanche” model (103). Based on his study, early HE was related to secondary multifocal micro- and macroscopic bleeding into the peri-hematoma area due to the ischemia and congestion following ICH (104). Several later studies (105, 106) and analyses of CT and SPECT in ICH (104, 107) added supportive evidences for the hypothesis, and the postulated mechanisms underlying these phenomenon include: (1) local tissue distortion caused by increased intracranial pressure; (2) blood brain barrier breakdown due to matrix metalloproteinase (MMP) activation; (3) secondary inflammatory reaction related to promoted activation, chemotaxis, and differentiation of macrophages, lymphocytes, microglia, and other inflammatory cells (108–111). However, it is difficult to verify the hypothesis without HE animal models.

## Medical Treatment For HE

Although the treatment of HE remains a challenge, researchers persevere in exploring effective solutions. At present, the treatment of HE is mainly divided into blood pressure control, hemostatic treatment, glucose management, and others.

## Intensive Blood Pressure-Lowering Treatment

Both INTERACT (112) and ATACH (113) have yielded safe and feasible intensive SBP lowering (≤140 mm Hg) in ICH. Moreover, subsequent INTERACT2 showed that intensive SBP lowering when obtained within the first hour and sustained throughout the first 24 h of ICH onset was related to reduced HE (114), specifically in basal ganglia ICH (15). However, the rate of mortality or disability was not decreased in intensive SBP lowering patients (14, 115), even in those with imaging predictors of HE (116, 117). This discrepancy may result from the neutralization effect that cardiorenal complications caused by intensive SBP lowering somehow diminished the benefits of suppressing HE (118, 119).

Substantial evidence has identified the presence of ischemic lesions both within and remote from the perihematoma region in ICH patients undergoing diffusion-weighted imaging (DWI) (120, 121). Intensive BP lowering may deteriorate the regional cerebral blood flow (CBF) after ICH and subsequently promote ischemic lesion formation. Studies have demonstrated that acute BP reductions after ICH is associated with decreased diffusion on DWI (122), and DWI-lesions are, in turn, correlated with poor functional outcomes (123). Thus, the presence of ischemic lesions may be a possible explanation for limited benefits after BP lowering in ICH patients.

Considering the potential side effects and yet improved functional outcomes of intensive BP reduction, the target goal of BP reduction, the optimal antihypertension drugs, and subgroup patients to the treatment are still problems. Recently target SBP of 130–139 mmHg has been suggested to be the optimal goal in the initial 24 h of acute ICH in the follow-up analysis of both INTERACT-2 (124) and ATACH-2 (119), but the support of larger randomized trials is lacking. Of note, in view of high BPV being associated with HE (20), administration of stable antihypertension drugs such as urapidil may result in better clinical outcomes and future studies should be conducted to compare different efficacies of antihypertension drugs in reducing HE. In addition, as not all ICH patients can benefit from intensive SBP lowering, it is necessary to select subgroups of patients at high risk of developing HE. Actually, studies have shown that those ICH patients who presents with an early onset, higher initial SBP, prior anti-thrombotic therapy, or milder neurological dysfunction at baseline were found to be associated with better functional outcomes in rapid SBP lowering (114, 125, 126). However, currently the characteristics of ICH patients benefiting from SBP control are still unclear and a complete screening scheme should be established to facilitate clinical practice in future studies.

To conclude, although intensive SBP lowering can reduce HE, not all patients can benefit from the therapy and further randomized clinical trials are needed to identify optimal patients for intensive SBP lowering treatment. Meanwhile, an appropriate target SBP level is essential for clinical decision-making and large prospective trials are needed to validate the proposed target SBP of 130–139 mmHg.

## Hemostatic Therapy

Platelet transfusion was once considered feasible for thrombocytopenia or antiplatelet medication-related ICH. However, platelet transfusion showed no overall benefit in ICH patients under antiplatelet treatment (127) and cannot be recommended due to its conversely higher mortality and insufficient efficacy at 3 months (128–132), because platelet transfusion cannot be completed within 6 h of onset when hemostasis is finished in most ICH patients (128). Moreover, recent studies have demonstrated that reduced platelet activity was related to early HE (133, 134) and was more common than reported prior antiplatelet therapy in ICH patients (135), thus improving platelet activity (such as desmopressin treatment) (136) after ICH has the potential to decrease HE, but further randomized trials are needed to verify the effect.

HE is more likely to occur in anticoagulant related-ICH and reversal agents might play an important role in the treatment of these patients. Compared with three-factor prothrombin complex concentrate (PCC) and fresh-frozen plasma (FFP) (137, 138), four-factor PCC, which might decrease HE, should be first recommended to reverse the effect of OAC with a more rapid international normalized ratio (INR) reduction, a better effect, and fewer adverse events (139–144). Studies investigating hemostatic therapy to reduce HE in NOAC-associated ICH are lacking, but several NOAC antagonists have been found. Idarucizumab, Andexanet Alfa, and PER977 have a demonstrated effect on reversal of NOAC dabigatran, FXa inhibitors, and edoxaban, respectively, (145–151), but their effect on HE needs to be further studied.

Mayer et al. conducted two successive trials, Factor Seven For Hemorrhagic Stroke-1, 2 (FAST-1, 2), both of which demonstrated recombinant factor VIIa (RFVIIa), can reduce HE but showed opposite results in clinical outcomes (152, 153). Later, meta-analyses of these and other clinical trials documented no effect on improving survival or functional outcomes after ICH (154). A recently published work also revealed that rFVIIa administration within 3 h from stroke onset cannot benefit spot sign-positive ICH patients (155). Notably, an excess of thromboembolic events remains a concern in RFVIIa treatment (156). Notwithstanding, subsequent subgroup analysis revealed that highly selected individuals, younger than 70 years old or hemophilia A patients, could benefit from RFVIIa infusion (157, 158).

Another tested hemostatic drug is tranexamic acid (TXA), which reversibly inhibits the conversion of plasminogen to plasmin. Tranexamic acid for hyperacute primary Intracerebral Hemorrhage trial (TICH2) is an international, randomized placebo-controlled trial aimed at evaluating the effect of TXA on ICH and has demonstrated that TXA could reduce HE, but functional outcomes at 90 days after ICH were not improved (9, 159). Moreover, increased risk of ischemic events remains a problem (160). Thus, a sub-study of TICH-2 which specifically aims at whether spot sign-positive patients could benefit from TXA administration is currently under way (161).

Several hemostasis therapies, including platelet transfusion, reversal agents of anticoagulants, and none specific hemostatic drugs such as RFVIIa and TXA, have been recommended for patients with hemostatic abnormalities to prevent HE in ICH. Although hemostasis therapy is an effective intervention for ICH-patients who have HE within the treatment window, its clinical practice is limited by conversely adverse effects from the long-term recovery. Moreover, in most cases hemostasis therapy only applies to those patients with coagulation dysfunction, and significant effect cannot be yielded from hemostasis therapy in hypertension-related ICH patients. Thus, future studies should pay attention to selecting the optimal ICH patients for hemostasis therapy to improve the efficacy.

## Glucose Management

Admission hyperglycemia is a predictor of HE independent of the presence of diabetes mellitus, and Qureshi et al. revealed that glucose monitoring and control in a rational range is correlated with the reduction of HE (83). However, intensive serum glucose lowering should not be recommended because of the increased risk of hypoglycemic events and even mortality in these patients (162–164). However, a preclinical study has reported that 17β-estradiol (E2) can attenuate hyperglycemia-related HE and improve neurological function in mice (165). Given that the increase of blood glucose is actually the consequence of energy metabolism dysfunction in the acute phase of ICH, the clinical value of short-term blood glucose control might be significant, but the target glucose level and the appropriate management to control glucose in ICH patients remains to be clarified.

## Others

A serials of preclinical researches of MMP inhibitors like CM352 (88, 166–168), and neuroprotectors such as NXY-059 (169, 170), NSP-116 (171), erythropoietin (172), valproic acid (173), memantine (174), curcumin (175), albumin (176), and tuftsin fragment 1-3 (177) have shed light on the treatment of HE. However, further studies to confirm the effect of CM352 and neuroprotectors are needed, since some pre-clinic medicines failed in clinical trials. For e.g., the Cerebral Hematoma And NXY Treatment trial (CHANT) has revealed that there is no effect of NXY-059 on the size of hematoma (170).

In conclusion, HE is a complex and dynamic process during ICH with poor functional prognosis. In consideration of the limited effect, we need to identify subgroup ICH patients who can benefit from the decrease of HE and therapies should give priority to those selected patients. Furthermore, individual characteristics such as age, hypertension history, intracranial pressure, and anticoagulant use should be taken into consideration when making decisions to treat HE. There is no appropriate animal model of HE, which limits the basic research and the discovery of new treatment target for HE. Thus, the discovery of a novel animal model which can mimic the natural process of ICH in humans would have a significant meaning.

## Author Contributions

ZL and MY was responsible for conception and design as well as initial drafting of the manuscript. CL and RB were responsible for revising the manuscript. HX was responsible for tables and figures. QH and BH read and approved the final manuscript.

## Conflict of Interest

The authors declare that the research was conducted in the absence of any commercial or financial relationships that could be construed as a potential conflict of interest.
